# LEA Detection and Tracking Method for Color-Independent Visual-MIMO

**DOI:** 10.3390/s16071027

**Published:** 2016-07-02

**Authors:** Jai-Eun Kim, Ji-Won Kim, Ki-Doo Kim

**Affiliations:** Department of Electronic Engineering, Kookmin University, Seongbuk-gu, Seoul 136-702, Korea; eun9477@kookmin.ac.kr (J.-E.K.); kjwon777@kookmin.ac.kr (J.-W.K.)

**Keywords:** visual-MIMO, GCM, color independency, LEA detection, LEA tracking

## Abstract

Communication performance in the color-independent visual-multiple input multiple output (visual-MIMO) technique is deteriorated by light emitting array (LEA) detection and tracking errors in the received image because the image sensor included in the camera must be used as the receiver in the visual-MIMO system. In this paper, in order to improve detection reliability, we first set up the color-space-based region of interest (ROI) in which an LEA is likely to be placed, and then use the Harris corner detection method. Next, we use Kalman filtering for robust tracking by predicting the most probable location of the LEA when the relative position between the camera and the LEA varies. In the last step of our proposed method, the perspective projection is used to correct the distorted image, which can improve the symbol decision accuracy. Finally, through numerical simulation, we show the possibility of robust detection and tracking of the LEA, which results in a symbol error rate (SER) performance improvement.

## 1. Introduction

Light emitting diodes (LEDs) are used in a wide variety of lighting equipment because they are eco-friendly, low-power devices. Recently, many studies of visual-multiple input multiple output (visual-MIMO) techniques have been conducted [1,2,3,4,5,6,7,8,9,10]. Here, visual-MIMO indicates visible light communication (VLC) between the light emitting array (LEA) and the camera. In this concept, optical transmissions by an array of light emitting devices are received by an array of photo detector elements (pixels) of a camera. The pixels in the camera can be treated as an array of highly directional receiver elements. This structure allows a reduction of interference and noise from other light sources in the channel. This system offers the freedom to select and combine a subset of receiver elements that receive a strong signal from the transmitter, and thus, achieves high signal-to-noise ratios (SNRs) [1]. In [3], the authors proposed an LED array detection method using M-sequence and an LED array tracking method using inverted signals. Here, an OOK modulated signal transmitted through LED array is received by a high-speed camera. In [5], LED array detection was performed using the pattern of Sync-LEDs. Based on Sync-LEDs, they could find out the start point of an on-off keying (OOK) modulated data sequence, and calibrate the distorted image snapshots. In [6], the communication between a vehicle and an LED traffic light was conducted using an LED traffic light as a transmitter, and an on-vehicle high-speed camera as a receiver. Here, the luminance value of LEDs in the transmitter should be captured in consecutive frame. In [7], a high frame rate CMOS image sensor camera was introduced as a potential V2I-VLC system. Also, inverted LED patterns were used for tracking. In [8], a special CMOS image sensor, i.e., an optical communication image sensor (OCI) was employed and a “flag image” obtained from OCI was used for real-time LED detection. In [9], they presented HiLight, a new form of real-time screen-camera communication without showing any coded images (e.g., barcodes) for off-the-shelf smart devices. Although it mentioned the importance of transmitter tracking, but a detailed analysis was not done, leaving the future challenges. In [10], we showed the applicability of generalized color modulation (GCM)-based visual-MIMO for V2X. By using the proposed visual-MIMO scheme, while performing seamless communication, we can maintain the original color and brightness in addition to increasing the capacity by using color encoding. As described above, in most of the previous works, a high-speed camera (or a special CMOS image sensor) was used as a receiver and an intensity-based modulation (e.g., OOK) has been used. On the other hand, we have used a general purpose commercial camera as a receiver. Also, in our paper, the detection and tracking method of an LEA optimized for GCM-based visual-MIMO is described systematically based on image processing. Our LEA detection method is best suitable for GCM and the GCM was originally proposed by ourselves [11]. This visual-MIMO based communication technique has numerous applications in situations in which line-of-sight (LOS) communication is desirable. For example, this approach enables novel advertising applications such as smartphone users pointing cell phone cameras at electronic billboards to receive further information including documents, movie clips, or website URLs. Another example is a museum application in which a kiosk display transfers exhibit information to cell phone cameras to produce maps, images, and customized audio museum tours. Applications are not limited to hand-held cameras and electronic displays: they also include vehicle-to-everything (V2X) communication, robot-to-robot communication, and hand-held displays for fixed surveillance cameras [8,10].

**Figure 1:**
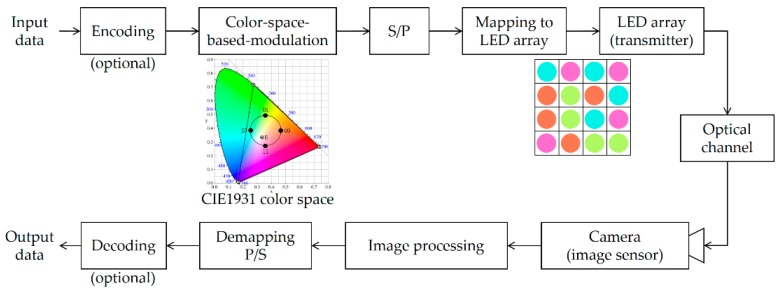
Color-space-based color-independent visual-MIMO transceiving procedure using image processing.

Additionally, LED lighting devices have been developed to a high level: currently, LEDs can emit light in various colors (close to full-color). The lighting color might be changed depending on the person’s emotion or environmental factors. However, it will be useful to achieve visible light communication that can maintain the original color and brightness while performing seamless communication. To solve this problem, a color-space-based modulation (CSBM) scheme, called generalized color modulation (GCM), was proposed and analyzed for color-independent VLC systems [11]. The modifier “color-independent” indicates the independence of the variations in the light color and light intensity. Some notable features of GCM include color independency, dimming control, and reasonable bit error rate (BER) performance during color variation. By incorporating GCM into visual-MIMO, we can obtain better symbol error rate (SER) performance, higher data rate over a larger transmission range, and most importantly, color independency when compared with conventional LED communication. Figure 1 shows a block diagram of the color-independent visual-MIMO method based on the color space [10].

At the transmitter, it performs the color-space-based modulation on the encoded data. Each constellation point in the light color space represents a corresponding color and a target color is the average of all appropriate constellation points. Here, target color indicates the wanted color of the LEA lighting. The target color corresponding to any VLC signal can be chosen from the gamut area and an information data stream can then be sent by choosing the appropriate constellation diagram corresponding to this target color. The proposed visual-MIMO system thus enables color-independent communication. Then, the serial to parallel conversion is performed on the modulated symbols by the LEA size. These symbols are then mapped onto LEA as prescribed order. At the receiver, a set of symbols (colors) are detected by the image sensor and the symbol (color) decision corresponding to each LED is performed by using image processing. Finally, the output of demapping in the color space is converted into a serial data stream that is sent to an information sink.

Because the camera is used as a receiver, a variety of distortions might occur when projected onto the image sensor, thereby adversely affecting the performance of color-independent visual-MIMO. In this paper, we propose an LEA detection and tracking method, shown in the image processing step in Figure 1, so as to improve SER performance. The remainder of this paper is organized as follows: Section 2 provides a detailed explanation of the proposed LEA detection and tracking techniques; Section 3 represents the results and discussion; finally, Section 4 concludes the paper.

## 2. LEA Detection and Tracking

**Figure 2:**
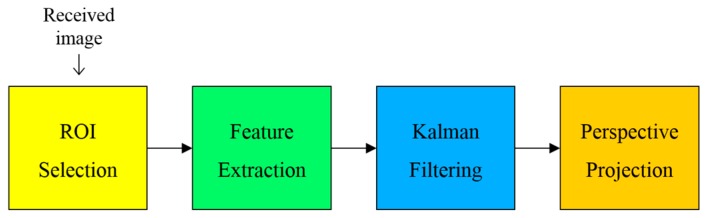
Block diagram of the proposed LEA detection and tracking method.

Although most of visual-MIMO related works [2,5,9] emphasized the importance of LEA detection and tracking, they did not address detail method and the corresponding effect on the communication performance. In [2,3,4], they did not consider the effect of misrecognition on the SER performance and also did not analyze the temporal movement of an LEA associated with tracking. On the other hand, in our paper, the detection and tracking method of an LEA optimized for color-independent visual-MIMO is described systematically based on image processing. Our proposed LEA detection (including tracking) method is also most suitable for the GCM to enhance the SER performance. In many practical applications of visual-MIMO (e.g., vehicle-to-vehicle (V2V) application), both the transmitter and the receiver can move. Therefore, we propose the LEA detection and tracking method shown in Figure 2.

**Figure 3:**
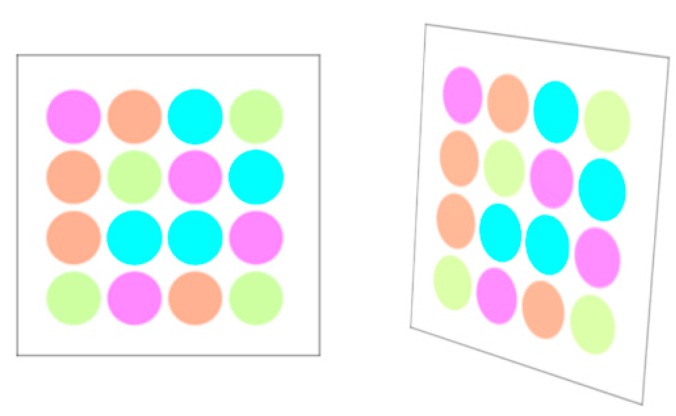
LEA configuration and shape distortion example of LEA on a received image.

The entire process consists of four steps. The first step is to specify the region of interest (ROI) for an LEA from the received image. Then, using the Harris corner detection algorithm [12], we extract the features of the LEA such as the corners of a rectangle. In the third step, a Kalman filter [13] is used to track the desired LEA. Finally, we correct the distorted shape of the LEA using perspective projection [14]. Figure 3 shows the original configuration and the distorted shape example of an LEA in the received image. As an experimental example, we used the LEA configuration of a square shape with a size of 4 × 4. The shape of the LEA on a received image can be distorted in the form of translation, rotation, and warping. We correct the distorted shape by utilizing the perspective projection technique, to increase the probability of right decision for each LED color (or symbol) in the array.

### 2.1. ROI Selection for LEA Detection

An image received by the camera is likely to include a complex background. In addition, the prominent features of LEA are that it has a rectangular shape and might also appear as a blob shape during LED light emission. However, these are very common features that other objects may also include. Therefore, it is not easy to detect the LEA over the entire image. To solve this problem, reference LEDs or reference LEA patterns can be used [3,5]. However, the use of references can lower the data rate and the appearance of the LEA might not be good.

**Figure 4:**
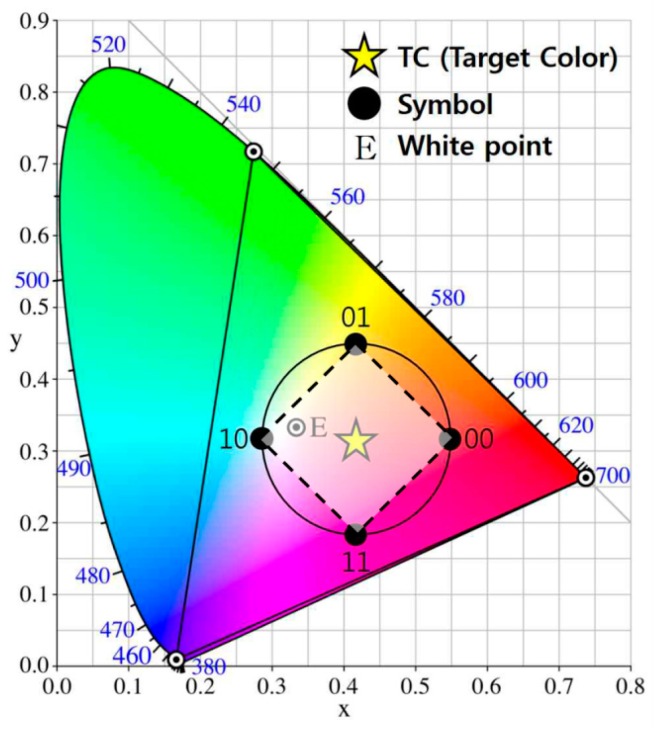
Example of circle-type constellation diagram in the CIE1931 color space.

To overcome this weakness, by using the principle of the color space, we specify the ROI for a desired LEA in a received image with a complex background. Because the color-independent visual-MIMO system uses a GCM that is based on a color space, the color information associated with the GCM can be an effective means of LEA detection. Figure 4 presents an example of a circle-type constellation diagram in the CIE1931 color space [15,16]. The input data symbol is represented by a constellation point (x, y) and each constellation point in the constellation diagram represents a color in the color space. In Figure 4, the target color, i.e., the color perceivable to human eyes after modulation, is the average of all appropriate constellation points. Here, constellation points in the color space can be arranged using a similar arrangement to that used in RF circular quadrature amplitude modulation (QAM). Supposing an equiprobable symbol transmission, which is reasonable, due to the compensation and interleaving algorithms, the target color can be obtained as the averaged RGB value along a number of symbols as shown in Equation (1) [10]:

(1) $$\left(x_{t},\;y_{t}\right)=\left(\frac{\sum_{i=1}^{N}x_{i}}{N},\;\frac{\sum_{i=1}^{N}y_{i}}{N}\right)$$

where $\left(x_{t},\;y_{t}\right)$ denotes the position of a target color and $\left(x_{i},\;y_{i}\right)$ denotes the position of the $i_{th}$ symbol. Note that the value of $\left(x_{t},\;y_{t}\right)$ is closer to the true target color when N is increasing in the probability sense. Here, N is the number of total LEDs of the LED array.

**Figure 5:**
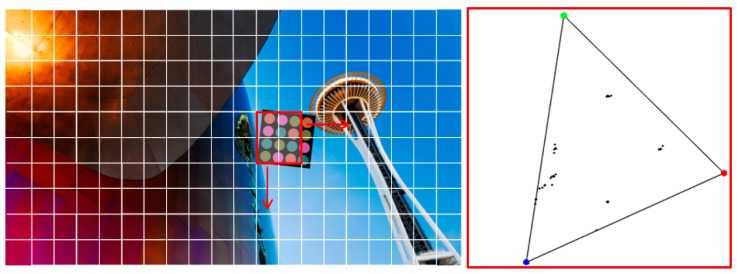
Received image example in the form of lattice structure and corresponding color distribution of pixels within the sliding search window area in the CIE1931 color space.

Figure 5 shows a received image example in the form of a lattice structure and the corresponding color distribution of the sliding search window area in the CIE1931 color space. In the experiment, we used a Windows 7 library image from Microsoft Corporation as a background. To select the ROI, we divided the image into a grid unit and used a sliding search window. The window consisting of four grids is indicated by the red box in Figure 5. This window is moved by the grid unit and the color distribution of the sliding search window area is analyzed in the CIE1931 color space to determine whether the desired LEA exists at that location. In a circle-type constellation diagram, it is important to note that the polygon formed by connecting the coordinate point of each symbol has a constant side ratio and symmetry property. Using these properties, in this paper, we propose an LEA detection method that analyzes the color distribution of a sliding search window area in a CIE color space. To classify the samples distributed in the two-dimensional color space, the *k*-means clustering algorithm is used [17]. Then, we select the desired ROI by checking the side ratio and the symmetry property of the polygon generated after the center of each cluster has been connected.

**Figure 6:**
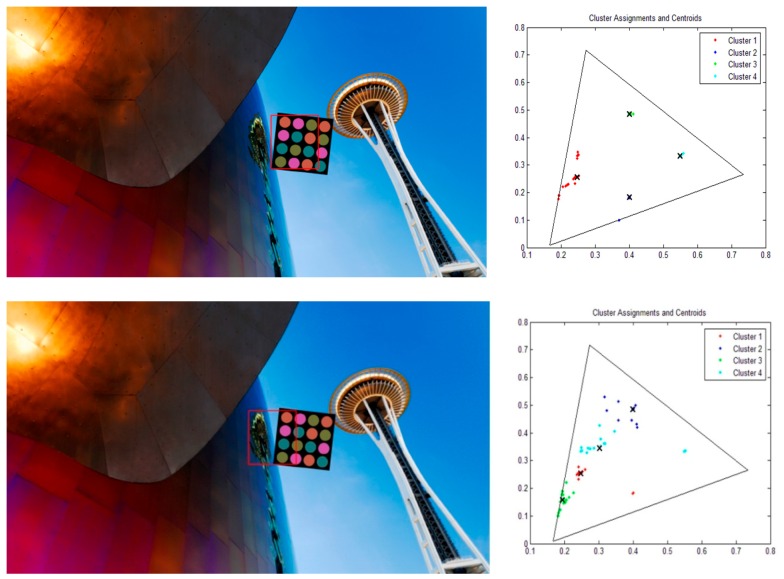

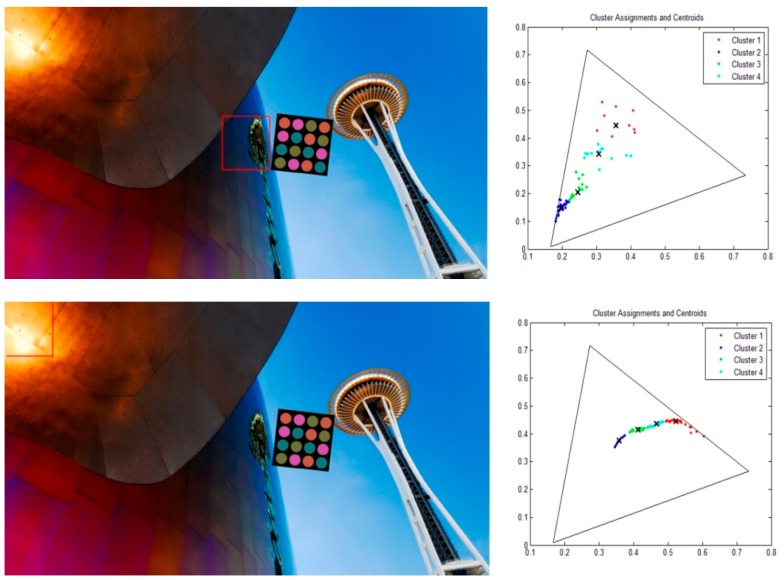
Examples of different locations of sliding search window and the corresponding color distribution of pixels within a window area in the CIE1931 color space.

Figure 6 shows examples for different locations of sliding search windows and the corresponding color distribution of a window area in the CIE1931 color space. We can see that the color is more uniformly distributed, when the window overlaps more with the desired LEA. To analyze the color distribution, we use the *k*-means clustering algorithm [17]. In these examples, the value of *k* is 4, and a quadrangle can be formed by connecting the center of each cluster. The ROI for the desired LEA can be selected if the aspect ratio value of a quadrangle is below the threshold.

### 2.2. LEA Detection Using the Harris Corner Method

The LEA configuration, as shown in Figure 3, has a rectangular shape and an intensity difference between the inside and the outside of the LEA. (This characteristic may be used to identify the LEA.) Because a quadrangle has four vertices, in this paper, we extract the vertices for LEA detection using the Harris corner method [12]. Harris corner method has been improved upon Moravec's corner detector by considering the differential of the corner score with respect to direction directly [12]. Here, the corner score is often referred to as the autocorrelation. For calculating the corner score, the sum of the squared differences is defined as in Equation (2):

(2) $$S\left(u,\;v\right)=\sum_{x,\;y}w\left(x,\;y\right){\left[I\left(x+u,\;y+v\right)-I\left(x,\;y\right)\right]}^{2}$$

where w(x, y) denotes the window at position (x, y), I(x, y) is the intensity at (x, y), and I(x+u, y+v) is the intensity at the moved window (x+u, y+v). Equation (1) can be expressed in matrix form as in Equation (3):

(3) $$S\left(u,\;v\right)=\left[u\;v\right]\left(\sum_{x,\;y}w\left(x,\;y\right)\left[\begin{array}{cc} I_{x}^{2} & I_{x}I_{y}\\ I_{x}I_{y} & I_{y}^{2} \end{array}\right]\right)\left[\begin{array}{c} u\\ v \end{array}\right]=\left[u\;v\right]\;M\;\left[\begin{array}{c} u\\ v \end{array}\right]$$

where $I_{x}$ and $I_{y}$ are the partial derivatives of *I*. Then, we can obtain the score for each window using Equation (4):

(4) $$S_{C}=\lambda_{1}\lambda_{2}-k{\left(\lambda_{1}+\lambda_{2}\right)}^{2}$$

where $\lambda_{1}$ and $\lambda_{2}$ are the eigenvalues of the matrix M in Equation (3), and *k* is a tunable sensitivity parameter. If $\lambda_{1}$ and $\lambda_{2}$ have large positive values, then the position (x, y) can be identified as a corner.

### 2.3. LEA Tracking with Kalman Filtering

The Kalman filter is used to estimate the location of the LEA in the received image when the relative position between the camera and the LEA varies. In addition, the smoothing effect of the Kalman filter improves the tracking result by reducing the uncertainty of the measurement noise [18]. Filtering also helps to handle the situation in which the corners of the LEA that are momentarily missed can be detected. For example, the LEA might be misrecognized as another similar square object or be obscured by obstacles.

**Figure 7:**
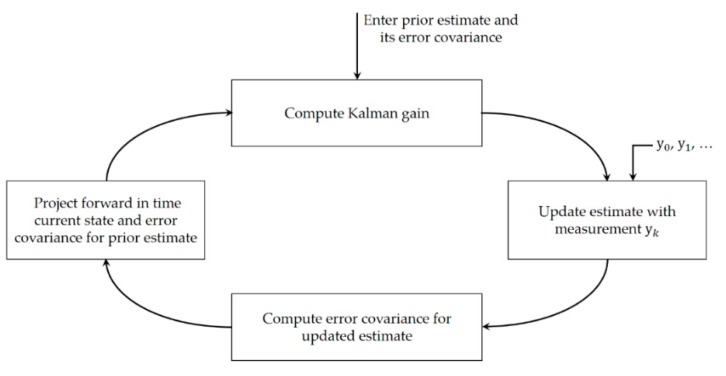
Discrete-time Kalman filter loop.

**Figure 8:**
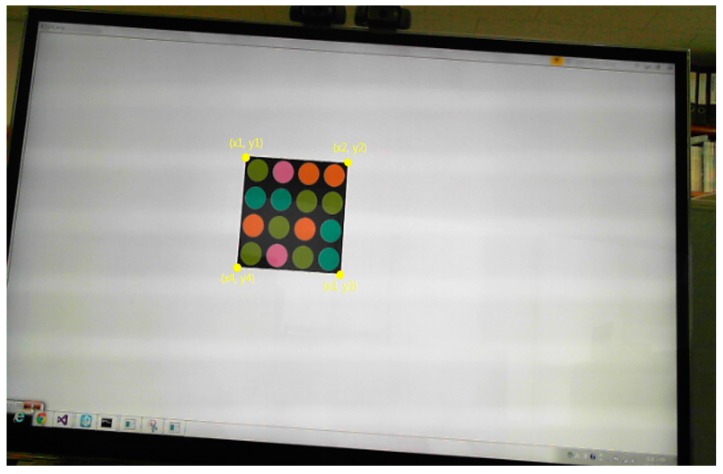
Four corners of LEA used as the variables of the Kalman filter.

We use a discrete-time Kalman filter to predict the motion of the LEA in the received image plane. To apply the Kalman filter to LEA tracking, we select the corner points of the LEA as the variables of the Kalman filter; therefore, the state vector $x_{k}$ and the measurement vector $y_{k}$ at time step *k* are defined as in Equation (5). Figure 7 presents a detailed overview of the discrete-time Kalman filter operation [13]. In Figure 8, we can see the four corners of the LEA, which are used as the variables of the Kalman filter.

(5) $$x_{k}=\left[x,\;y,\;v_{x},\;v_{y}\right]y_{k}=\left[x,\;y\right]$$

### 2.4. Perspective Projection for Correcting Image Distortion

If LEA detection and tracking are successfully implemented, we must then make a symbol (color) decision for each LED. Because the camera is used as a receiver, the received image can be distorted, which adversely affects the SER performance. The distorted shape of the LEA is not suitable for determining the symbol for each LED inside the square LEA using image processing. To correct the distorted shape of the LEA, perspective projection is used in the final step of our proposed method, as shown in Figure 2. Geometrical distortions such as scaling, rotation, skewing, and perspective distortion are very common transformation effects. Each distortion is represented by a linear transformation, which is well-investigated in linear algebra [19]. These transformations can be performed using simple multiplication, as shown in Equation (6):

(6) $$\left(\begin{array}{ccc} a_{1} & a_{2} & b_{1}\\ a_{3} & a_{4} & b_{2}\\ c_{1} & c_{2} & 1 \end{array}\right)\times \left(\begin{array}{c} x\\ y\\ 1 \end{array}\right)=\left(\begin{array}{c} x^{\prime }\\ y^{\prime }\\ 1 \end{array}\right)$$

where $\left(\begin{array}{cc} a_{1} & a_{2}\\ a_{3} & a_{4} \end{array}\right)$ is a rotation matrix. The above matrix defines the kind of transformations that will be performed: scaling, rotation, and so on. $\left(\begin{array}{c} b_{1}\\ b_{2} \end{array}\right)$ is the translation vector; it simply moves the points in the *x*-*y* plane. $\left(\begin{array}{cc} c_{1} & c_{2} \end{array}\right)$ is the projection vector. Here, *x* and *y* are the source points on the received image. And *x’* and *y’* are the destination points of the transformed coordinates.

**Figure 9:**
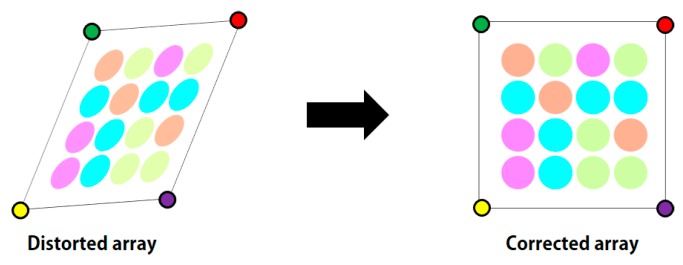
Perspective projection to correct the distorted array.

Finally, given the four corner points of the LEA in the image, the perspective projection can be applied to correct the distorted LEA, as shown in Figure 9.

## 3. Results and Discussion

In an actual experiment, a commercial camera (Logitech's webcam) was used as a receiver and the resolution of a received image is 640 × 480 (VGA resolution). We used the Open CV library to implement our proposed algorithm. Table 1 shows the parameters of a transmitter used in the simulation. The size of the LEA is 4 × 4 and the number of symbols (or constellation points) is four. We transmit a total of 16,000 symbols to ensure the reliability of the received performance result and compute the SER. We used the CIE1931 color-space-based constellation shown in Figure 4.

**Figure 10:**
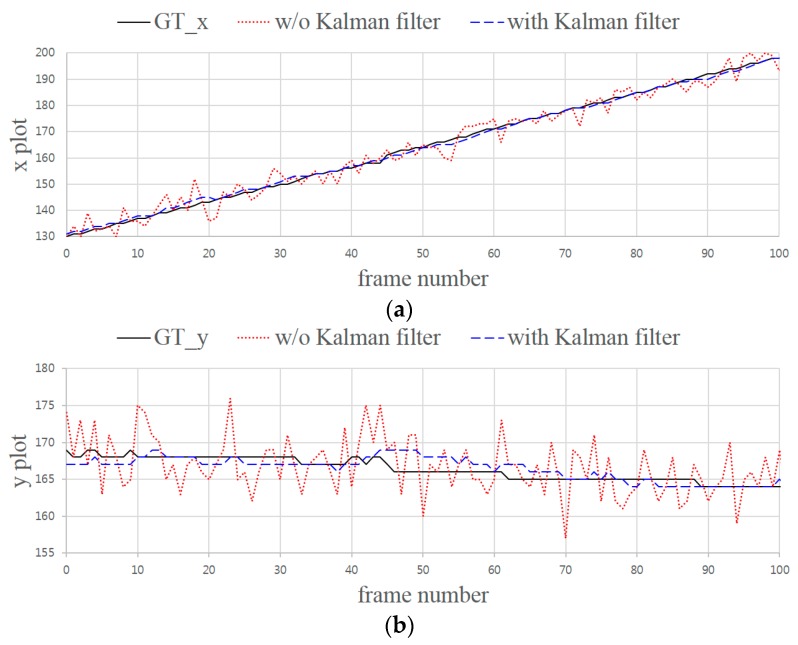
Tracking results for a horizontally moving LEA. (**a**) *x* plot vs. frame number; (**b**) *y* plot vs. frame number

In our simulation, we assumed that the LEA on the display device moved horizontally with a constant velocity and that color distortion did not occur during transmission. We designedly added only a Gaussian error value to the points of the corners. Therefore, we consider only errors that occur in the process of projection as a target region for the symbol decision. Figure 10 shows the tracking results for a horizontally moving LEA. The centroid of the LEA was tracked over 100 frames to verify the performance of the Kalman filter. In the figure, “GT” indicates the ground truth, which represents the true location of the moving LEA. From Figure 10a, it can be seen that the LEA is moving from left to right. From Figure 10b, it also can be seen that the LEA moves horizontally, because the vertical movement is very small. The results show that the position of the moving LEA can be tracked better when the Kalman filter is applied.

**Figure 11:**
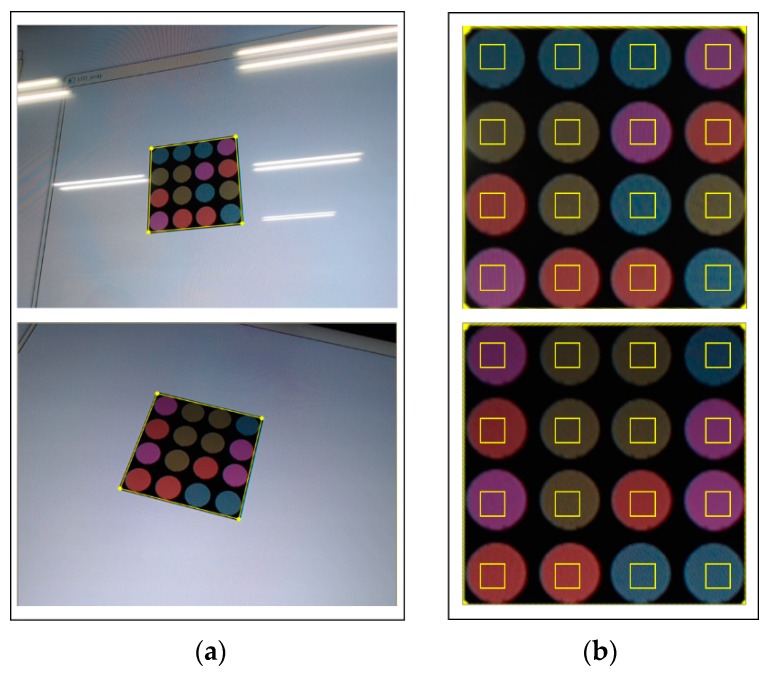
Perspective projected results for the detected LEA. (**a**) Distorted received image; (**b**) Perspective projected results.

Figure 11 shows the correction results for the detected LEA after perspective projection. In the figure, the small yellow box inside the LED represents the area used for the symbol (or color) decision. Although LEA detection and correction were successfully performed, the symbol (or color) decision might be difficult because of image distortion, which causes the yellow box to be placed outside the LED region. However, if perspective projection is performed, the decision area is determined correctly and the SER performance can be improved.

**Figure 12:**
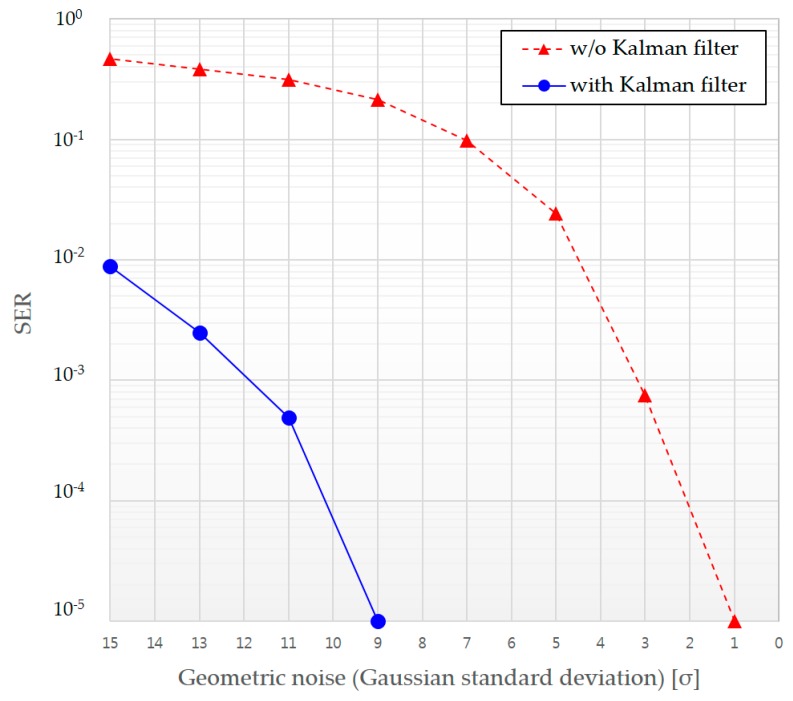
SER performance comparison with and without Kalman filtering.

Figure 12 presents the improvement in SER performance when we use a Kalman filter with perspective correction.

## 4. Conclusions

In this paper, we proposed an LEA detection and tracking method for a color-independent visual-MIMO system utilizing image processing technique that is applicable to various applications. To increase the reliability of LEA detection, we selected the ROI using color-space-based analysis of the received image with a complex background. Next, the LEA with a square shape was detected using the Harris corner method. Furthermore, we used a Kalman filter to better track the moving LEA, which can be disturbed by obstacles. Finally, to facilitate the color (or symbol) decision for each LED, the perspective projection process was performed on the distorted image. Experimental results show that our proposed method provides reliable LEA detection and tracking. Furthermore, SER performance is improved when using perspective projection with a Kalman filter.

## Figures and Tables

**Table 1:** Simulation parameters.

Parameter	Value
Color space	CIE1931
RGB model	CIE RGB
Reference white	E
LED array size	4 × 4 (16)
Number of constellation points	4
Intensity (Y value)	0.165
Total number of symbols transmitted	16,000
Three positions of RGB LEDs in the CIE1931 space	R: (0.0735, 0.265)
	G: (0.274, 0.717)
	B: (0.167, 0.009)

## Supplementary Materials

The following are available online at http://www.mdpi.com/1424-8220/16/7/1027/s1. Figure S1: The illustration of LEA Detection and Tracking Method for Color-independent Visual-MIMO.

## Abbreviations

The following abbreviations are used in this manuscript:

BER	bit error rate
GCM	generalized color modulation
CSBM	color-space-based modulation
LEA	light emitting array
LED	light emitting diode
LOS	line-of-sight
MIMO	multiple-input multiple-output
ROI	region of interest
SER	symbol error rate
SNR	signal-to-noise ratio
V2V	vehicle-to-vehicle
V2X	vehicle-to-everything
VLC	visible light communication
