# Supplementary material for: LEA Detection and Tracking Method for Color-Independent Visual-MIMO
Source: Sensors (Basel). 2016 Jul 2;16(7):1027. doi: 10.3390/s16071027 (PMC4969837; doi:10.3390/s16071027)
Supplement: Supplementary file 1 [file sensors-16-01027-s001.pdf]

Sensors **2016**, *16*, 1027; doi:10.3390/s16071027

Supplementary Materials: LEA Detection and Tracking Method for Color-Independent Visual-MIMO

Jai-Eun Kim, Ji-Won Kim and Ki-Doo Kim

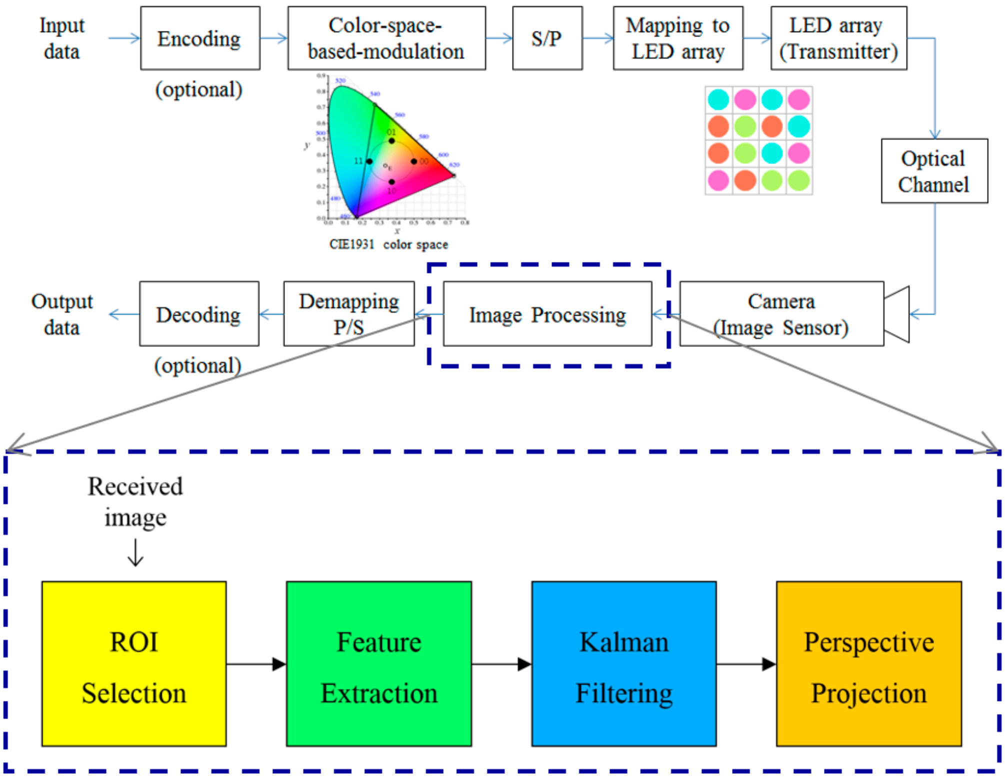

Figure S1. The illustration of LEA detection and tracking method for color-independent visual-MIMO.
